# Glycation damage to organelles and their DNA increases during maize seedling development

**DOI:** 10.1038/s41598-022-06454-7

**Published:** 2022-02-17

**Authors:** Diwaker Tripathi, Delene J. Oldenburg, Arnold J. Bendich

**Affiliations:** Department of Biology, University of Washington, Seattle, WA USA

**Keywords:** Biochemistry, Cell biology, Developmental biology, Molecular biology, Plant sciences

## Abstract

Shoot development in maize begins when meristematic, non-pigmented cells at leaf base stop dividing and proceeds toward the expanded green cells of the leaf blade. During this transition, promitochondria and proplastids develop into mature organelles and their DNA becomes fragmented. Changes in glycation damage during organelle development were measured for protein and DNA, as well as the glycating agent methyl glyoxal and the glycation-defense protein DJ-1 (known as Park7 in humans). Maize seedlings were grown under normal, non-stressful conditions. Nonetheless, we found that glycation damage, as well as defenses against glycation, follow the same developmental pattern we found previously for reactive oxygen species (ROS): as damage increases, damage-defense measures decrease. In addition, light-grown leaves had more glycation and less DJ-1 compared to dark-grown leaves. The demise of maize organellar DNA during development may therefore be attributed to both oxidative and glycation damage that is not repaired. The coordination between oxidative and glycation damage, as well as damage-response from the nucleus is also discussed.

## Introduction

DNA damage can be caused by agents originating outside of the cell, such as radiation, and agents created within the cell, such as reactive oxygen species (ROS) generated during the electron transport processes in respiration and photosynthesis. Regardless of how DNA damage arises, that damage must be repaired in the diploid nucleus because the cell needs both copies of the nuclear genome to function properly. The integrity of chromosomal DNA molecules in the nucleus is generally well-maintained during development from germline to adult tissues, as is expected from more than a hundred years of cytological study of chromosomes. However, the same cannot be said for chromosomal DNA molecules in the plastids (ptDNA) and mitochondria (mtDNA), where molecular integrity varies substantially among plant species, with maize representing the most extreme case of DNA degradation^1^. The most sensitive assays of DNA integrity have been the analysis of isolated and ethidium-stained DNA molecules in moving pictures and pulsed-field gel electrophoresis. The organellar DNA (orgDNA; ptDNA and mtDNA) molecules in maize are generally larger than the size of the genome for basal meristem tissues but highly degraded for green leaves. The fragmented molecules also carry unrepaired damage that inhibits DNA polymerase in long qPCR assays, and some of that damage can be rectified by added DNA repair enzymes *in vitro*^2^.

Several reasons could account for the difference in molecular integrity between nuclear DNA and orgDNA. (i) DNA repair systems are more numerous in the nucleus than cytoplasmic organelles^1,3–5^. (ii) Although there are many “copies” of the organellar genomes in diploid cells, the high metabolic activity in the organelles (i.e., respiration and photosynthesis) creates biochemical conditions that produce byproducts that can damage orgDNA. Thus, some or most of these “copies” may not be capable of encoding functional gene products even though copy-number assays such as blot-hybridization and real-time qPCR report such defective DNA molecules as copies^1^. (iii) A damaged copy or fragment of orgDNA may serve in signaling genotoxic stress to the nucleus^5–7^, which could then modulate DNA repair activities throughout the cell.

Most research on the repair of DNA damage created within the eukaryotic cell has focused on oxidative damage. Recently, we reported 8-hydroxydeoxyguanosine (8-oxoG) damage in maize orgDNA and found that it increased during development, as well as being higher in light- compared to dark-grown leaves^7^. However, there is another type of damage caused by the glycation of nucleic acids, proteins, and small molecules (see below). Glycation involves the non-enzymatic covalent addition of a reactive carbonyl group from sugars or the small-molecule glycolytic by-products such as methylglyoxal [CH_3_–CO–CHO; MGO] and glyoxal [CHO–CHO; GO] to amino and thiol groups^8,9^. Among the nucleotides in human DNA, deoxyguanosine (dG) is the most reactive in glycation, and the MGO-adduct dG-MG is five times more prevalent than the most common oxidative adduct (8-oxoG)^10^.

Glycation proceeds through a series of intermediates resulting in Advanced Glycation End-products (AGEs), and glycation damage is thought to contribute at least equally with oxidative damage to DNA damage in cells^10,11^. A recent study showed that the protein DJ-1 and its bacterial homologs could repair methylglyoxal- and glyoxal-glycated nucleotides and nucleic acids (DNA and RNA) and that depletion of DJ-1 led to increased levels of glycated DNA, DNA strand breaks, and strong mutator phenotypes^11^. Thus, DJ-1 repairs guanine glycation damage to maintain DNA integrity. Another report showed that human DJ-1 also repairs methylglyoxal- and glyoxal-glycated cysteines, arginines, and lysines as free amino acids and in proteins^12^. The dual role of DJ-1 makes it the only enzyme known to repair both proteins and nucleic acids.

**Figure 1:**
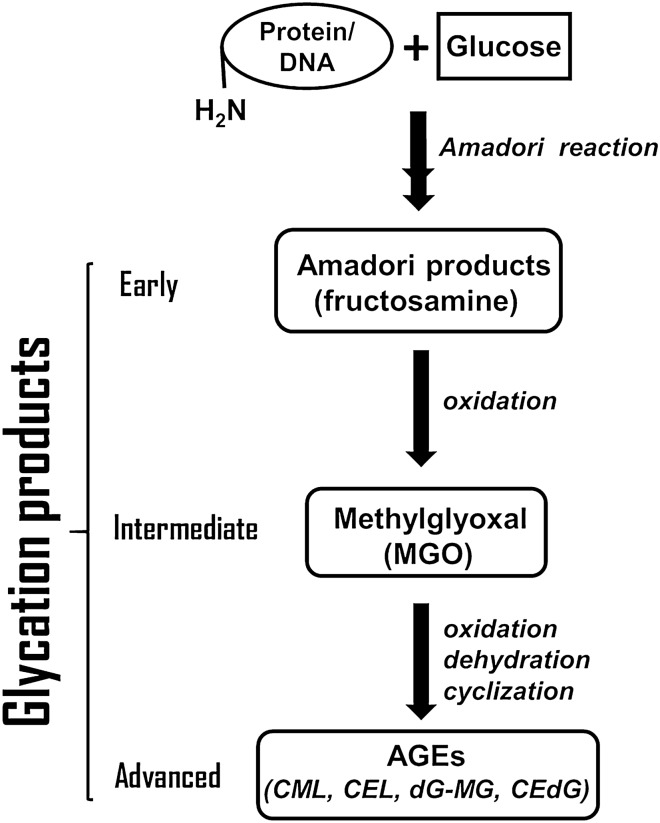
Key steps in glycation process. Reducing sugars interact with amino groups of protein or nucleic acids to form unstable Schiff bases that undergo intramolecular rearrangement to form Amadori products (early glycation products) such as fructosamine. These products are converted to reactive dicarbonyl compounds (intermediate glycation products) such as methylglyoxal (MGO), glyoxal, and 3-deoxyglucosone that are then irreversibly converted into advanced glycation products (AGEs). All the reactions are spontaneous (not enzymatically catalyzed). Further details are provided in refs 10, 14, and 46. Examples of AGE adducts to proteins and DNA: CML: Nε-(carboxymethyl)-lysine; CEL: Nε-(carboxyethyl)-L-lysine; CEdG: N2-(1-carboxyethyl)-deoxyguanosine; dG-MG: 3-(2′-deoxyribosyl)-6,7-dihydro-6,7-dihydroxy-6-methylimidazo-[2,3-b]purine-9(8)one.

The glycation process leading to irreversible AGE adducts to proteins and DNA is biochemically complex, and a few key steps of DNA glycation damage are shown in Fig. 1. The damage can be ameliorated in two ways: (i) detoxifying the small-molecule aldehydes in a glyoxalase damage-avoidance process; or (ii) removing the glycated adducts on DNA in a deglycase-repair process^13^. Repair/avoidance of glycation damage is conducted by a family of glyoxalase proteins, such as Parkinson’s disease related Park7 protein^14^, and family members are present probably in all organisms, including all land plants surveyed^15^. In rice and maize, the nuclear genome contains genes for 11–12 members of the DJ-1 group of glyoxalases, and some of these DJ-1 proteins are known or predicted to be targeted to mitochondria and plastids^15^. Homologs of DJ-1/Park7 are present in Arabidopsis, and AtDJ-1a (At3g14990), AtDJ-1b (At1g53280) and AtDJ-1c (At4g34020) are 35%, 41% and 35% similar in amino acid sequence to human DJ-1/Park7, respectively^16^. Among Arabidopsis DJ-1 proteins, AtDJ-1a protects the plant from excess light by promoting antioxidant activity. AtDJ-1b is a redox-sensitive sulfenylated protein found in the chloroplasts and mitochondria^17^. AtDJ-1c was found to be essential for plastid development, with more of this protein found in young leaves than in older leaves^18^. Most studies on glycation in plants have focused of glycation of proteins. To our knowledge there have been no reports about glycation damage in organelles and their DNA.

As light-grown maize plants develop from the meristem at the base of the shoot to mature leaves, the size of orgDNA decreases from molecules equal to or greater than the size of the genome in the base of the stalk (meristem) to much smaller fragments in the leaf^1,2,19^. In dark-grown plants, however, high-integrity ptDNA (large complex-branched molecules) is retained in the leaves. A rapid decline in ptDNA copy number is observed after transfer from dark to light growth conditions^20^. In our present study, we investigate potential changes in glycation damage during development and under light or dark growth conditions that may lead to the demise of orgDNA.

We previously reported on changing levels of ROS, antioxidant agents, and DNA damage in developing mitochondria and plastids of maize^7^. Here, we do the same for glycation damage and glycation defense because we suspect that glycation and oxidative damages in plant organelles may be related. We find that glycation damage, as well as countermeasures against glycation, follow the same developmental pattern found for ROS: as damage increases, damage defense measures decrease. In addition, we find less glycation damage in seedlings grown in the dark compared to light. The cells appear to reduce their “effort” to defend against both oxidative and glycation damage as promitochondria and proplastids develop into mature mitochondria and chloroplasts.

## Results

Here, we report changes in the levels of glycation adducts in proteins and DNA, as well as MGO and DJ-1, in organelles at three stages of maize development: Stalk lower (the base of the stalk), Stalk upper (top of the stalk), and the blades from the first three leaves. Assays were also performed using leaves from dark-grown and light-grown seedlings. In maize, L1 refers to the first and oldest leaf, while L2 and L3 refer to the second and third leaves. We used equal amounts of organelles, total protein or DNA isolated from organelles in our assays (see “Methods”).

### AGE protein level increases during leaf development

**Figure 2:**
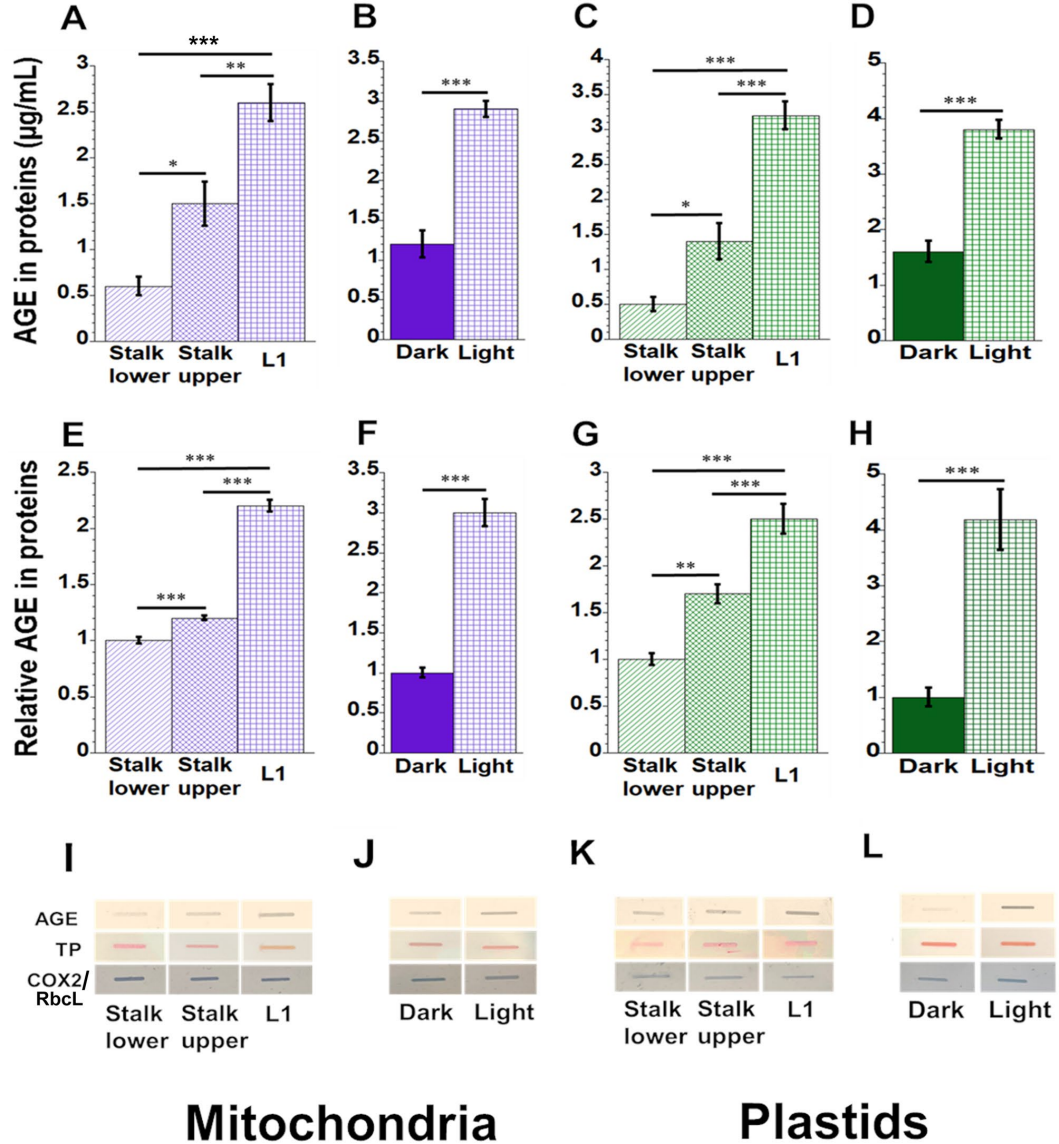
AGE in maize mitochondria and plastids. The amount of AGE in proteins extracted from isolated mitochondria and plastids was measured using anti-AGE antibodies with a competitive ELISA (**A**–**D**) and slot blot (**E**–**L**) assays. For the ELISA assay the levels of AGE in total mitochondrial and plastid proteins were measured as µg/mL. Representative images of slot blot assays are shown in (**I–L**) and the relative AGE in protein was determined, where the image intensity was set to 1 for stalk lower in (**E**, **G**) and for dark-grown leaves in (**F**, **H**). Uppermost panels of (**I**–**L**) show AGE proteins, middle panels show the total protein (TP) levels in samples, and lowermost panels show mitochondrial (COX2)- and plastid (RbcL)-specific proteins (COX2 in panels I and J; RbcL in panels K and L). Equal amounts of protein extracted from the different tissues were used in these assays. Data here and in Figs. 3, 4, 5, 6 are represented as mean ± SEM. Statistically significant differences were measured using ANOVA statistic test with post hoc analysis using Tukey’s HSD and are shown as asterisks, where *P-value ≤ 0.05, **P-value ≤ 0.01, ***P-value ≤ 0.001 are indicated on respective graphs.

To measure glycated proteins in different maize tissues, we isolated organelle proteins from leaf and stalk tissues. Our data showed a difference in glycation damage in mitochondrial and plastid proteins as measured using anti-AGE antibodies with ELISA and slot-blot assays (Fig. 2). In mitochondria, a fourfold increase in AGE protein glycation was found as Stalk lower develops to Stalk upper and then to fully developed L1 leaf (Fig. 2A), while the AGE level was almost 2.5 times higher in mitochondrial protein from light-grown leaves than dark-grown leaves (Fig. 2B). In plastids, proteins from L1 had approximately six times the AGE level than Stalk lower (Fig. 2C), while plastids from light-grown leaves had 2.4-fold more AGE than dark-grown leaves (Fig. 2D). Similar results were obtained with slot-blot assays where the relative intensity of AGE proteins was twofold higher in mitochondria from L1 than from Stalk lower (Fig. 2E) and light-grown leaves contained threefold more AGE proteins than dark-grown leaves (Fig. 2F). For the slot-blot assay with plastids, the level of AGE proteins was 2.5-fold higher in plastids from L1 than Stalk lower (Fig. 2G). Also, light-grown leaf plastids had 4 times the level of AGE proteins than plastids from dark-grown leaves (Fig. 2H). Representative images of slot blots for different mitochondrial and plastids samples are shown in Fig. 2I-L.

### DJ-1 levels in organelles decline during leaf development

**Figure 3:**
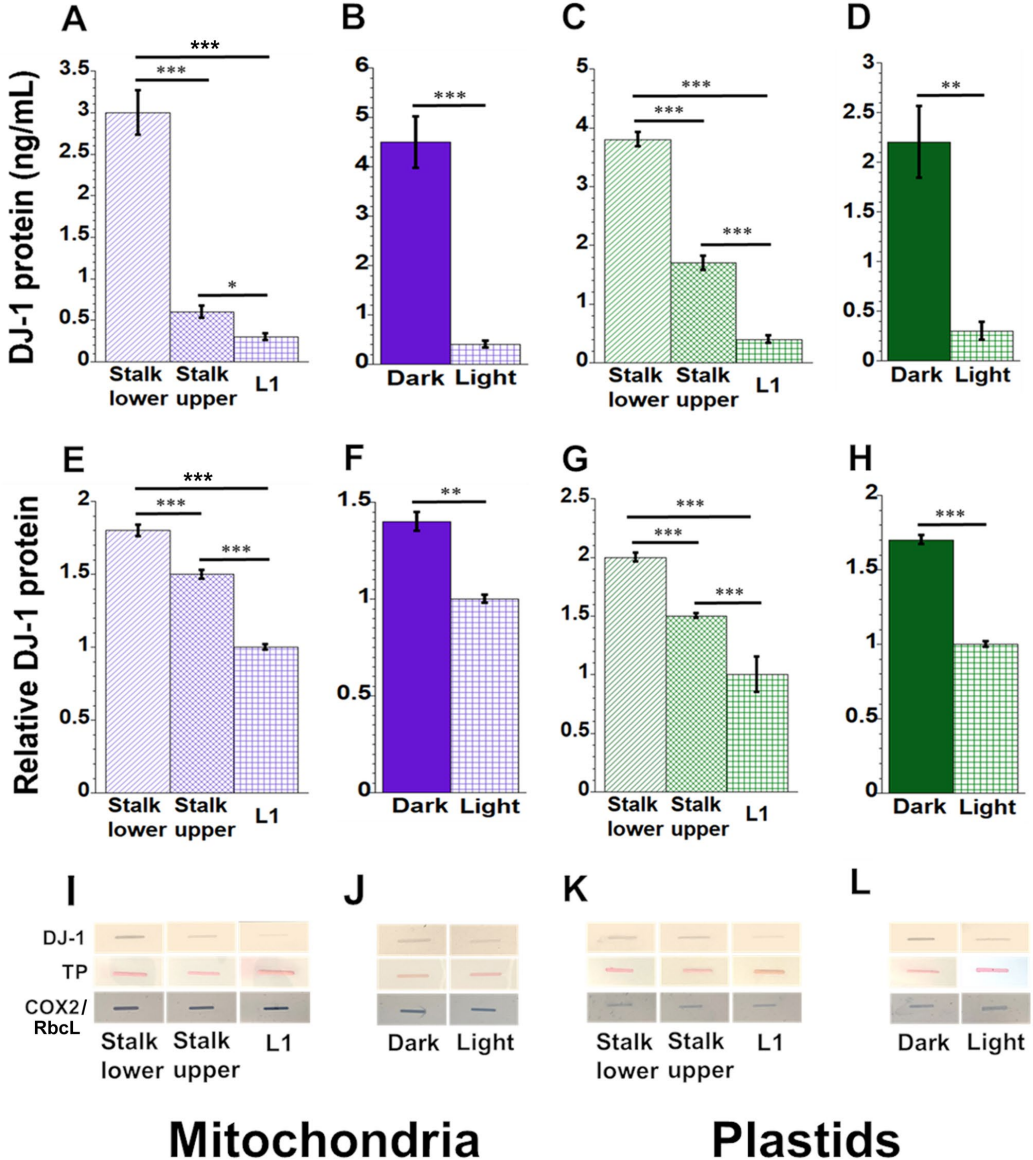
DJ-1 protein in maize mitochondria and plastids. The amount of DJ-1 protein from isolated mitochondria and plastids was measured using anti-DJ-1 antibodies with competitive ELISA (**A**–**D**) and slot blot (**E**–**H**) assays. For ELISA, DJ-1 protein in mitochondria was measured as ng/mL. Representative images of slot blot assays are shown (**I**–**L**) and the relative DJ-1 protein was determined, where the image intensity was set to 1 for leaf 1 (L1) in (**E**, **G**) and for light-grown leaves in (**F**, **H**). Uppermost panels of I-L show DJ-1 proteins, middle panels show the total protein (TP) levels in samples, and lowermost panels show mitochondrial (COX2)- and plastid (RbcL)-specific proteins (COX2 in panels I and J; RbcL in panels K and L). Cross-reactivity of maize organellar DJ-1 with anti-DJ-1 antibodies (human Park7) was confirmed and is shown in Supplementary Information, Fig. S1.

DJ-1 acts as an antioxidant and to ameliorate glycation damage in most organisms^13,21^. Endogenous levels of DJ-1 protein in maize mitochondria and plastids were determined using human anti-DJ-1 antibodies with ELISA and slot-blot assays. To confirm the cross-reactivity of human anti-DJ-1 antibodies, a western blot was performed with mitochondria from maize and Arabidopsis. The human DJ-1 antibodies recognized several homologs of DJ-1 proteins in plants. Interestingly, we found more DJ-1 bands from the dark-grown maize leaf mitochondria compared to light-grown leaf mitochondria, suggesting the presence of more than one form of DJ-1 (Supplementary Information, Fig. S1). In our slot blot assays, the level of DJ-1 protein in mitochondria was tenfold lower in fully developed L1 than in Stalk lower (Fig. 3A). Furthermore, the DJ-1 level was 11-fold lower in mitochondria isolated from leaves grown in the light than grown in dark (Fig. 3B). DJ-1 protein levels in plastids showed the same trend as shown in mitochondria. By ELISA, the DJ-1 level was almost 9 times lower in plastids isolated from L1 than Stalk lower (Fig. 3C). The DJ-1 levels from plastids isolated from leaves grown in light were 7 times lower than that grown in the dark (Fig. 3D). Similar results were found with the slot-blot assays: the DJ-1 protein level was 1.8-fold less in mitochondria isolated from L1 than Stalk lower tissue (Fig. 3E) and 1.4-fold less in mitochondria from light-grow than dark-grown leaves (Fig. 3F). In slot blot assays (Fig. 3G,H), the relative DJ-1 level in plastids was 2 times lower in L1 than Stalk lower tissue, and the level from leaves grown in light was 1.7-fold lower than that grown in the dark. Representative images of slot blots for each mitochondrial and plastid sample are shown in Fig. 3I-L.

In summary, despite the increase during the development of glycation damage to proteins in mitochondria and plastids (Fig. 2), the production of the glycation-defense protein DJ-1 decreases for both mitochondria and plastids (Fig. 3).

### Methylglyoxal levels vary during organelle development

**Figure 4:**
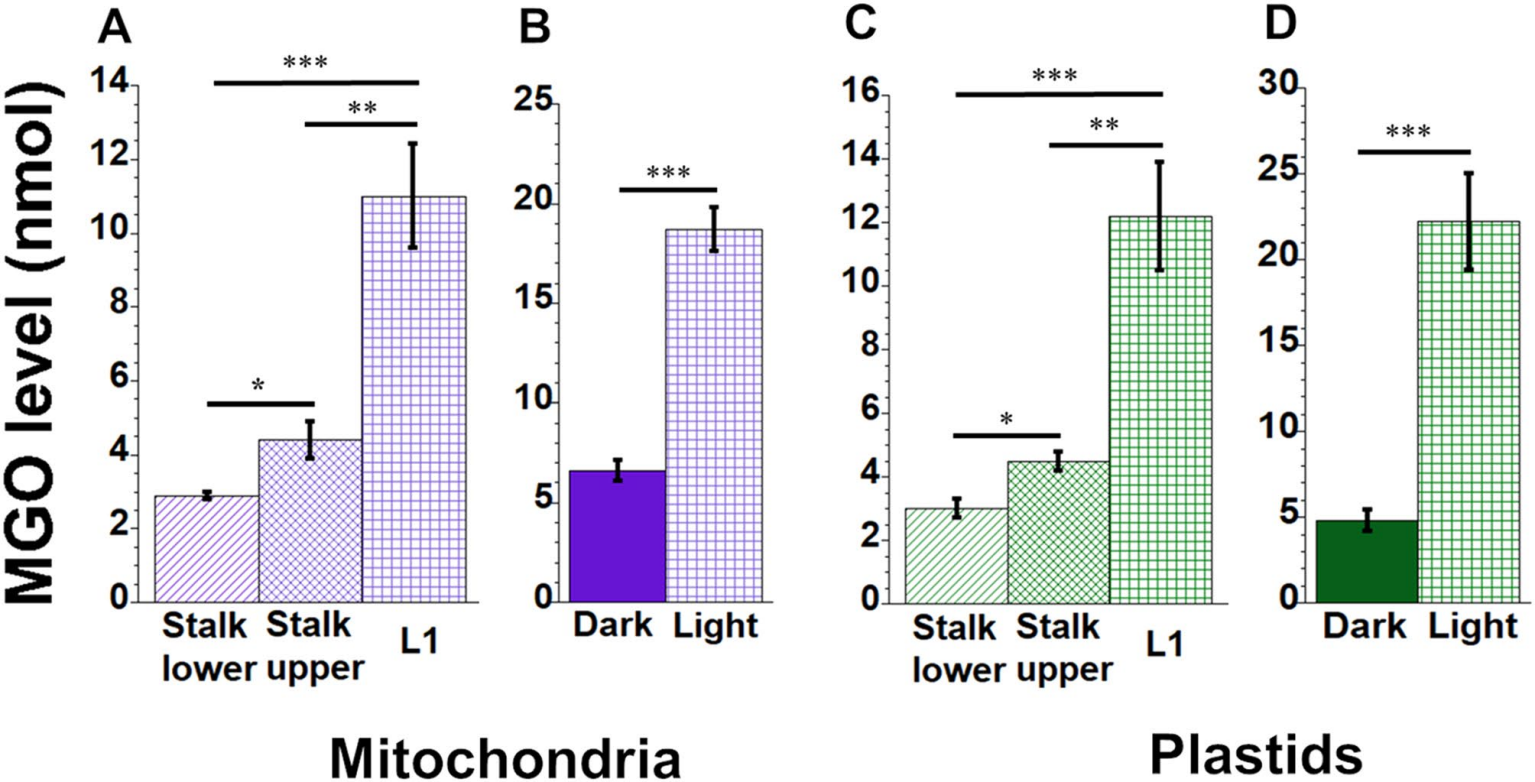
Methylglyoxal (MGO) in maize organelles. Mitochondria and plastids were isolated and MGO levels in nmole determined with a MGO assay kit as per manufacturer’s instructions. (**A** and **B**) show MGO in mitochondria; (**C** and **D**) show MGO in plastids.

Since MGO is generated within cells from glucose and amino acid metabolism and ketone oxidation, we hypothesized that MGO levels might vary during leaf development. To measure MGO levels in mitochondria and plastids, we used an MGO assay kit (Abcam). This assay measures total MGO in organelles, including free or soluble MGO as well as MGO adducts to proteins and DNA. The mitochondria from L1 had a 3.7-fold higher level of MGO than Stalk lower (Fig. 4A), and mitochondria from light-grown leaves had 2.8-fold more MGO than dark-grown leaves (Fig. 4B). Similarly, plastids from L1 had 4.1-fold more MGO than Stalk lower (Fig. 4C), and light-grown leaves had 4.8-fold more MGO than dark-grown leaves (Fig. 4D).

### Levels of Amadori products in organellar DNA vary during development

**Figure 5:**
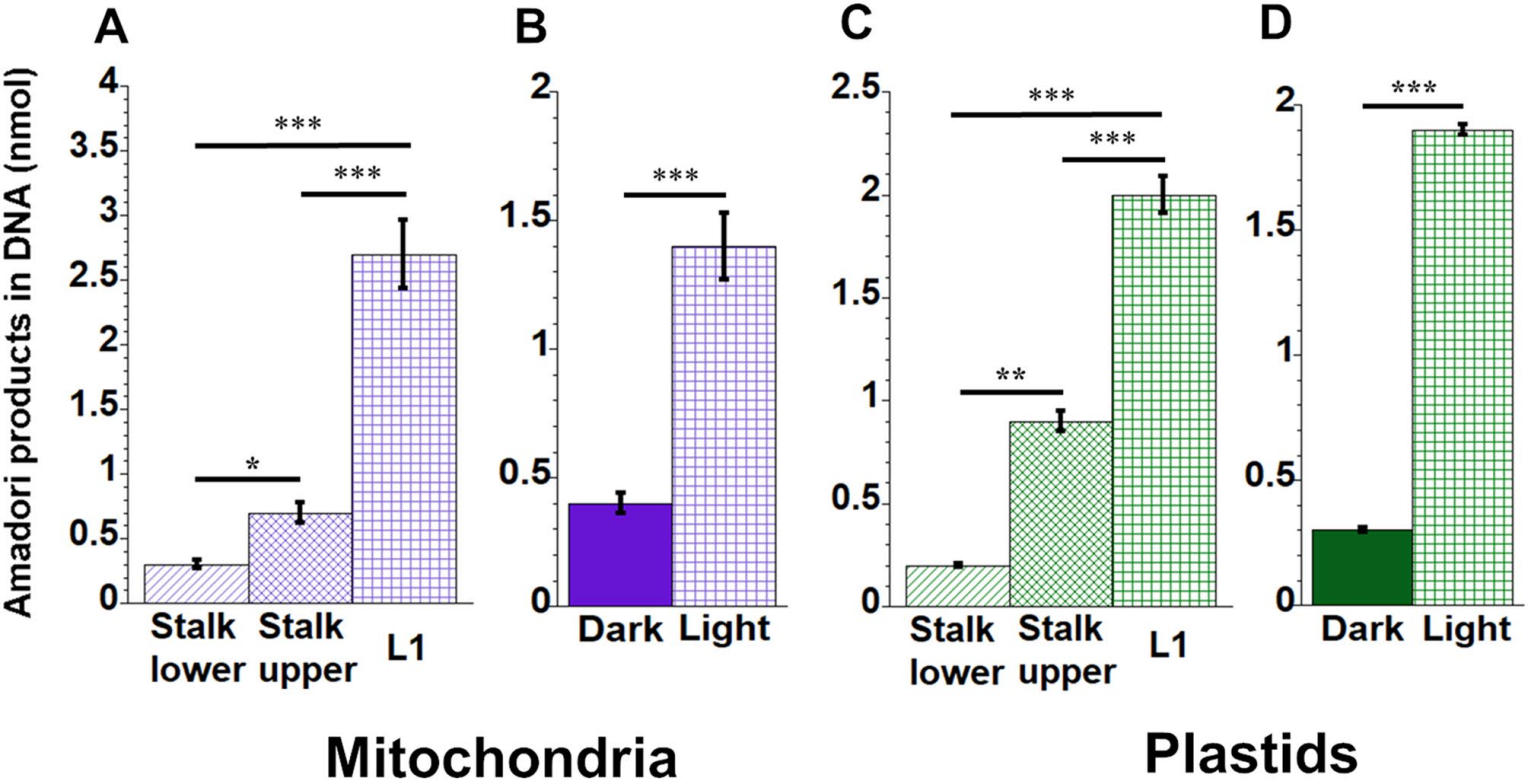
Amadori products in maize organellar DNA. MtDNA (**A** and **B**) and ptDNA (**C** and **D**) were incubated with nitro-blue tetrazolium for 5 h at 37 °C. The absorbance of fructosamine and its level in nmole were determined. Equal amounts of mtDNA or ptDNA extracted from the different tissues were used in these assays.

Glycation of proteins and DNA occurs at early and advanced stages. An early step involves forming Amadori products, such as fructosamine, that later may form products known as AGEs (Fig. 1). In order to monitor glycation damage to organellar DNA, we assessed the level of fructosamine in orgDNA, using the nitro-blue tetrazolium chloride (NBT) reduction assay^22,23^. Our data showed a ninefold increase of the fructosamine content in mtDNA for L1 compared to Stalk lower (Fig. 5A), and the fructosamine content was 3.5-fold greater for light-grown than dark-grown leaves (Fig. 5B). The NBT assay with ptDNA showed a similar trend (Fig. 5C): a ten-fold higher fructosamine content in ptDNA for L1 than for Stalk lower. Additionally, the fructosamine content in ptDNA was sixfold higher for light-grown leaves than ptDNA from dark-grown leaves (Fig. 5D).

### Levels of AGE in orgDNA increase during development

**Figure 6:**
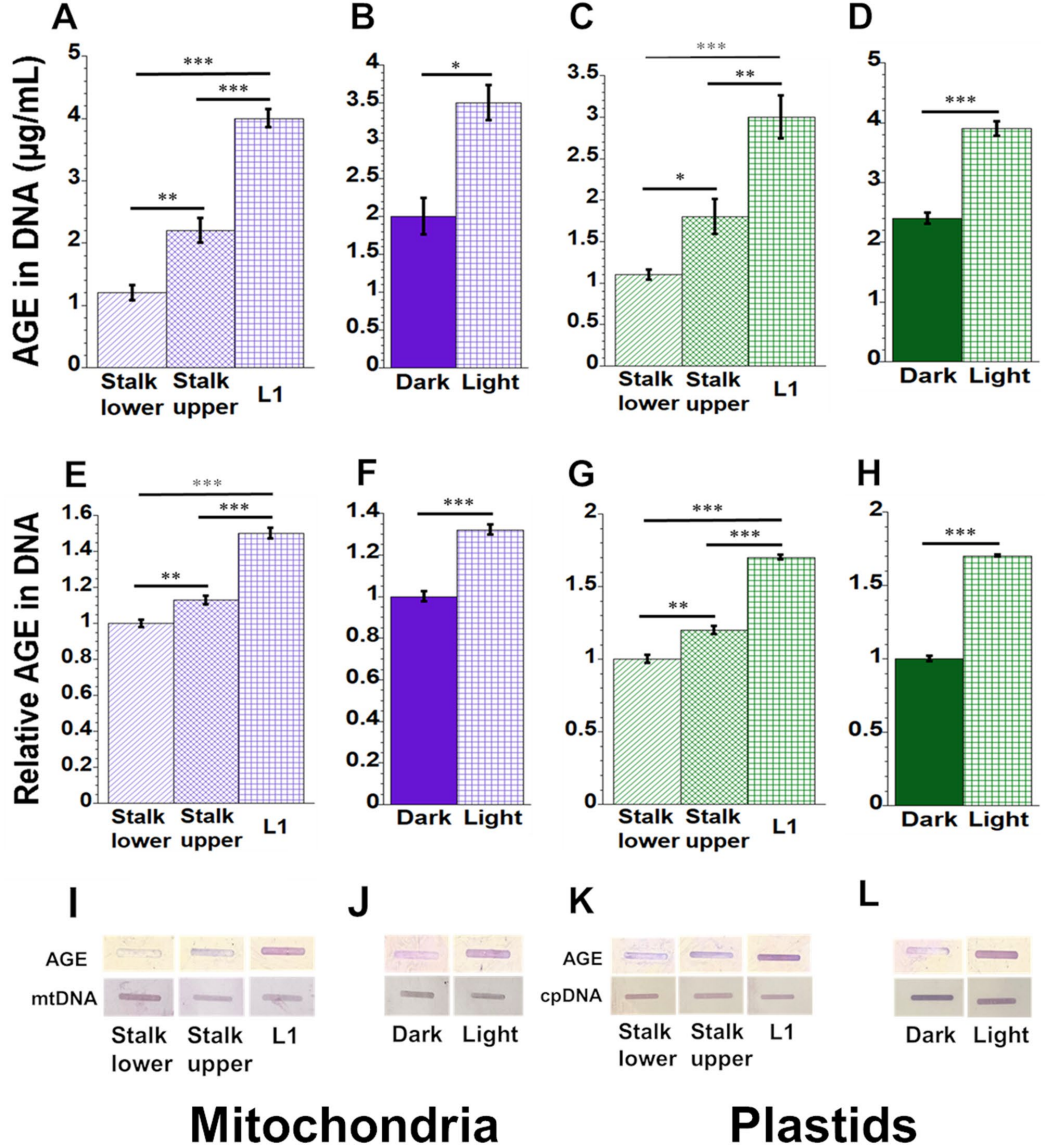
AGE in DNA isolated from mitochondria and plastids. For competitive ELISA assays (**A**–**D**), AGE levels were determined as µg/mL. The relative levels of AGE were also determined by slot blot assays (**E**–**H**), and representative images are shown (**I**–**L**) where the image intensity was set to 1 for Stalk lower in (**E**, **G**) and for dark-grown leaves in (**F**, **H**). Equal amounts of mtDNA extracted from the different tissues were used in these assays. Lower panels of (**I**–**L**) show the equal amount of orgDNA as determined by slot blot using mitochondrial- and plastid-specific probes (cox2 and rbcL, respectively).

To assess glycation damage in maize, we isolated mtDNA and ptDNA from developing leaf and stalk tissues and probed with antibodies to AGE adducts. We used two different assays to assess the AGE level in these DNAs, competitive ELISA, and slot-blot, both of which employ anti-AGE antibodies. We found 3.5 times as much AGE attached to mtDNA isolated from L1 than from Stalk lower (Fig. 6A), and the AGE level was 1.7 times higher in mtDNA isolated from light-grown than dark-grown leaves (Fig. 6B). For plastids, there were 2.7 times more AGE residues attached to ptDNA isolated from L1 than from Stalk lower (Fig. 6C) and 1.6 times more AGE residues for light-grown than dark-grown leaves (Fig. 6D), as determined with the ELISA assay. We found similar results from the slot-blot assay where the relative intensity of the AGE in DNA was 1.5-fold higher in mtDNA from L1 than from Stalk lower (Fig. 6E). Similarly, the light-grown leaves had 1.3 times more AGE in DNA than dark-grown leaves (Fig. 6F). In the plastid slot-blot assay, the relative level of AGE residues attached to ptDNA was 1.7-fold higher in L1 than in stalk lower (Fig. 6G); and for light-grown leaves, it was 1.7 times higher than for dark-grown leaves (Fig. 6H). Representative images of mtDNA and ptDNA slot blots are shown by Figs. 6I-L.

Overall, we found that the levels of glycation (early, intermediate, and end) products increase, while the levels of glycation-defense protein DJ-1 decrease during leaf development. These changes result in increasing glycation damage to the proteins and DNA within mitochondria and plastids as the leaf develops from the basal meristem. We also found more glycation damage and less DJ-1 in light-grown than in dark-grown leaves, suggesting protection against DNA damage in non-photosynthetic etioplasts.

## Discussion

We previously reported that ROS levels in mitochondria, plastids, and whole cells increased during maize development from the basal meristem to the green leaf, whereas the levels of antioxidant agents (such as peroxidases, glutathione, and ascorbate) declined during development^7^. Here, we show that another type of molecular damage, glycation damage, as well as the level of the glycation-defense protein DJ-1, follow the same developmental pattern found for ROS: damage increases, and damage-defense measures decrease (Table 1). A unifying theme emerges that the DNA and molecules that attend DNA are actively preserved in the germline, where damage-associated respiration and photosynthesis do not occur, and repair costs are low probably because germline cells carry the information for future generations. As the cells differentiate to build the adult plant to support reproduction (a single growing season for maize), the organelles and their DNA are minimally repaired until the increasingly defective DNA molecules and organelles are removed, probably by autophagy.

**Table 1:** Summary of results from the assays.

	Mitochondria	Plastids
AGE protein level	Leaf^1^ (2–4 times) > stalk lower; Light^2^ (2.5–3 times) > Dark^3^	Leaf (2.5–6 times) > stalk lower; Light (2.4–4 times) > Dark
DJ-1 protein level	Stalk lower (1.8–10 times) > Leaf; Dark (1.4–11 times) > Light	Stalk lower (2–9 times) > Leaf; Dark (1.7–7 times) > Light
MGO level	Leaf (3.7 times) > stalk lower; Light (2.8 times) > Dark	Leaf (4.1 times) > stalk lower; Light (4.8 times) > Dark
Amadori products level	Leaf (9 times) > stalk lower; Light (3.5 times) > Dark	Leaf (10 times) > stalk lower; Light (6 times) > Dark
AGE DNA level	Leaf (1.5–3.5 times) > stalk lower; Light (1.3–1.7 times) > Dark	Leaf (1.7–2.7 times) > stalk lower; Light (1.6–1.7) > Dark

^1^Leaf (L1).

^2^Total leaves (L1 + L2 + L3) grown in light.

^3^Total leaves (L1 + L2 + L3) grown in dark.

### Perception and response to damage during development

How can we account for the strikingly similar reciprocal relationship between damage and damage-defense during leaf development for ROS and glycation? Since this relationship applies to both mitochondria and plastids of maize, we may gain insight from previous work with mitochondria in mammals. de Bari et al*.*^24^ concluded that oxidative stress and MGO are interconnected and in equilibrium with each other and that high levels of mitochondrially generated ROS can lead to an increase in MGO, AGEs, and cell death. In cultured mouse cells deficient in superoxide dismutase, glycation of mtDNA (but not nuclear DNA) indicated to Breyer et al.^25^ that oxidative stress promotes glycation of mtDNA. They proposed that H_2_O_2_ degrades deoxyribose, creating reactive carbonyl species that react with guanine (G) to yield CEdG, a DNA-bound AGE that they measured by competitive ELISA. Another report showed that methylglyoxal reacts with lysine in a glycation reaction to generate superoxide and hydrogen peroxide that eventually cause DNA damage^26^. During maize development, we found increasing oxidatively damaged mtDNA and ptDNA^7^ and increasing AGEs in mtDNA and ptDNA using NBT (Fig. 5) and competitive ELISA (Fig. 6) assays. Thus, it appears that for orgDNAs, oxidative and glycation damage and damage-response are coordinated in plants and animals.

Comparisons between light- and dark-grown leaves also provide useful information about orgDNA damage and repair responses. In dark leaves high orgDNA copy number and integrity are maintained in preparation for the transition from etioplast to photosynthetically active chloroplast upon exposure to light, which subsequently induces a rapid decline in orgDNA^1,20,27^. Previously, we found less orgDNA damage and greater defenses against ROS-induced damage in dark-grown leaves^2,7^ and now show a similar trend—lower DNA glycation damage and higher DJ-1 levels.

Both kinds of damage (ROS and glycation) may be perceived on a cell-wide basis, according to the following scenario. Among the various types of ROS, H_2_O_2_ is the least chemically reactive but the most able to cross membrane boundaries^28–30^. Regardless of where in the cell ROS is produced (mitochondria, plastids, peroxisomes), the “signal” (either H_2_O_2_ or a protein it mobilized^31^) reaches the nucleus announcing the redox status of the cell. In a meristematic cell of maize, the nucleus responds to the weak signal (low H_2_O_2_) by delivering DNA repair proteins to the promitochondria and proplastids^7^. In differentiated cells, however, the signal strength increases, and the nucleus responds by reducing the expression of repair proteins targeted to the mature mitochondria and chloroplasts, leading to the increasing damage we observe during maize development. Data for Arabidopsis are also consistent with the abandonment of glycated ptDNA, although the degradation of ptDNA during plastid development is less rapid and less extensive in Arabidopsis than in maize^32,33^. Lin et al. concluded that DJ1C, a member of the DJ-1 superfamily, functions mainly in the early stages of differentiation of proplastids to etioplasts and chloroplasts^18^. Whereas full-strength repair in the diploid nucleus must continue because both copies are needed, the organelles have many copies of their DNA and can afford to lose most of them.

### Defense against glycation damage in mitochondria and plastids

The glycating agents MGO and GO are produced spontaneously during sugar metabolism and lipid peroxidation, so that all organisms need to defend against the ensuing damage^34,35^. The main pathway of MGO detoxification is the glyoxalase system comprised of glyoxalases 1 and 2 (I and II in plants) and glutathione^36,37^. Some organisms, including plants, also have glyoxalase III enzymes (DJ-1 is one of these) that do not require glutathione^15^, and our data show that DJ-1 is inversely correlated with damage to mtDNA and ptDNA (Figs. 3 and 6). DJ-1 is reported to have additional functions beyond glycation defense^13^**,** including acting as an antioxidant by scavenging ROS^37^. Transgenic tobacco plants expressing Hsp31 (a yeast homolog of DJ-1) were tolerant of a variety of *imposed* stresses, including H_2_O_2_ stress, that raised MGO levels in whole leaf cells and redirected Hsp31 to mitochondria^34^. Our data for maize show that DJ-1 levels decrease in mitochondria and plastids isolated from leaves developing normally (without imposed stress) and in light versus dark growth conditions (Fig. 3). Higher DJ-1 levels in organelles from the stalk and dark-grown tissues suggest that DJ-1 is protecting these organelles from glycation as well as oxidative damage.

### Damaged and undamaged “copies” of organellar DNA

Organellar genome copy number per diploid cell is large and highly variable during plant development, whereas the copy number in the nucleus of the diploid cell remains essentially constant throughout development. Nonetheless, orgDNA-encoded proteins persist at fixed molar ratios with their nuclear DNA-encoded subunit partners in multi-subunit protein complexes, such as ribosomes, cytochrome oxidase, and RUBISCO. This conundrum has been appreciated for decades, and applies to plants, animals, and protists^38^. For bacteria in the wild, it is essential for survival to be able to flexibly and rapidly respond to a changing environment, and the genome must be intact and in supercoiled form to do so^39^. The organellar genomes are descendant from bacteria and probably retain that “intactness” requirement for coordinated expression among genes separated by small and large distances. The basal meristem of maize includes cells with intact orgDNA molecules destined to carry the germline, whereas in green leaves there are no detectable genome-sized orgDNA molecules, only highly degraded linear fragments not in supercoiled form^27,40^. Thus these fragmented and damaged molecules probably cannot respond properly to environmental or developmental cues that require gene expression to be “coordinated” in response to signals, such as H_2_O_2_.

The copy number conundrum (also known as the “ploidy paradox”) may be attributed to three factors: (i) inadequate methods for estimating genome copy numbers that score defective versions of orgDNA as useful copies^1,7^; (ii) failure to appreciate the requirement for intact, supercoiled genome-sized molecules for optimal cellular performance; and (iii) failure to appreciate that abandonment of orgDNA may be a biochemical cost-savings stratagem for high-copy genomes found in mitochondria and plastids when low-damage metabolism switches to respiration and photosynthesis^41,42^. The developmental changes we now report for glycation of organellar DNA and protein (which should include DNA-repair proteins) should reduce the number of functional genome copies among the number previously scored as “copies.”

## Methods

### Plant tissue and growth conditions

Maize [*Zea mays* (L.), inbred line B73] seeds were originally obtained from the Maize Genetics Cooperation Stock Center Catalog of Stocks at Agricultural Research Service (https://data.nal.usda.gov/dataset/maize-genetics-cooperation-stock-center-catalog-stocks) and then propagated annually in our departmental plant facility. As described previously^7,40^, seeds were imbibed overnight and sown in Sunshine soil Mix #4 and vermiculite (1:1 ratio). The seedlings were grown for 12 days with a 16 h light/8 h dark photoperiod (light-grown) or in continuous dark for 12 days (dark-grown) in a temperature-controlled room. The light intensity was ∼500 μmol s^–1^ m^–2^ photosynthetic photon flux density (PPFD). Seedlings were washed with 0.5% sarkosyl for ∼3 min and then rinsed with distilled water. For each assay, tissue was harvested from 30 to 50 plants as described previously^7^. Briefly, the seedlings were divided as follows: Stalk lower (base of Stalk 5 mm above the node); Stalk upper (top of Stalk 5 mm below the ligule of the first leaf), leaf blades (L1 or L1 + L2 + L3). Stalk tissue was comprised of several concentric rings of leaves, the outermost being the first leaf sheath. L1 was the fully expanded blade, whereas L2 and L3 were still developing.

### Isolation of organelles (plastids and mitochondria)

Organelles were isolated using high-salt buffer [HSB; 1.25 M NaCl, 40 mM HEPES pH 7.6, 2 mM EDTA pH 8, 0.1% BSA, 0.1% β-mercaptoethanol (Sigma-Aldrich)] as described in previous reports^7,27,40^. Briefly, tissues were homogenized in HSB, and differential centrifugation was applied to pellet mitochondria and plastids. The homogenate was differentially centrifuged first at low speed (500×g for 5 min) to remove nuclei. Then the supernatant was centrifuged (3000×g for 10 min) to pellet plastids. The resulting supernatant was centrifuged at 20,000×g for 15 min) to pellet mitochondria. The plastid and mitochondria pellets were washed three times with chloroplast dilution buffer (CDB; 0.33 M D-sorbitol, 20 mM HEPES pH 7.6, 2 mM EDTA, 1 mM MgCl_2_, 0.1% BSA) and mitochondria dilution buffer (MDB; 0.4 M D-sorbitol, 0.1 M HEPES pH 7.6, 2 mM EDTA, 1 mM MgCl_2_, 0.1% BSA), respectively. The organelles were further purified using discontinuous (step) Percoll gradients as follows. For plastids, 30% and 70% Percoll solutions adjusted to the equivalent osmolality of 1 × CDB were prepared (for example: for 30%, 12 mL Percoll + 8 mL 5 × Chlp Gradient Buffer + 20 mL dH_2_O and for 70%, 28 mL Percoll + 8 mL 5 × Chlp Gradient Buffer + 4 mL dH_2_O; 5 × Chlp Gradient Buffer is 1.65 M D-sorbitol, 40 mM HEPES pH 7.6, 4 mM EDTA, 2 mM MgCl_2_, 0.2% BSA). For two-step gradients, 15 mL 30% Percoll was layered onto 15 mL of 70% Percoll in a 40-mL centrifuge tube. Then 2–4 mL of plastid solution was gently layered on top, followed by centrifuged for 30 min at 1500×g using a JA-20 fixed-angle rotor. Plastids were removed from the 30/70 Percoll interface, transferred to a centrifuge tube and washed 2–3 times with CDB (using 10 × the volume of recovered plastid solution), followed by centrifugation of 3000×g for 8 min to pellet plastids. The purified plastids were then resuspended in a small volume of CDB. A similar process was used for purification of mitochondria, except for the following minor changes. A two-step 28% and 45% Percoll gradient, with solutions adjusted to the equivalent osmolality of 1 × MDB, was used (for example: for 28%, 11.2 mL Percoll + 20 mL 2 × Mito Gradient Buffer + 8.8 mL dH_2_O and for 45%, 18 mL Percoll + 20 mL 2 × Mito Gradient Buffer + 2 mL dH_2_O; 2 × Mito Gradient Buffer is 0.8 M D-sorbitol, 40 mM HEPES pH 7.6, 4 mM EDTA, 2 mM MgCl_2_, 0.2% BSA). Centrifugation was done for 20 min at 20,000×g using a JA-20 fixed-angle rotor, mitochondria were recovered from the 28/45 Percoll interface, washed 2–3 times with MDB, pelleted by centrifugation for 15 min at 20,000×g, and resuspended in small volume of MDB. Finally, the purified mitochondria and plastids were resuspended and saved in a small volume of MDB or CDB, respectively. Freshly isolated organelles were used in the assays.

### Isolation of total proteins from the organelles

The organellar proteins were isolated as described^43^ with some modifications. Plastid and mitochondrial pellets were mixed with protein extraction buffer [50 mM HEPES, 1% CHAPS, 1X HALT protease inhibitor cocktail (Thermo Fisher Scientific)]. The mixture was vortexed for 5 min at high speed and was centrifuged at 10,000×g for 10 min. The supernatant was collected, and total protein amount was quantified with a Pierce™ BCA Protein Assay kit (Thermo Fisher Scientific).

### Isolation of DNA from organelles

PtDNA and mtDNA were extracted using cetyltrimethylammonium bromide (CTAB) with some modifications^44^. An equal volume of 2 × CTAB buffer [2% CTAB (w/v), 100 mM Tris/HCl (pH 8.0), 20 mM EDTA, 1.4 M NaCl, 1% polyvinylpyrrolidone (Mr 40,000; w/v); preheated to 65 °C and Proteinase K (20 μg/ml)] were added to the resuspended plastids or mitochondria and incubated at 65 °C for 1 h. Then 0.1 M phenylmethylsulfonyl fluoride was added, followed by incubation at room temperature for 1 h. Then RNase A was added to 100 μg/mL, and the samples were kept at 60 °C for 15 min. Next, potassium acetate was added to 400 mM, and the mixtures were kept on ice for 15 min before centrifugation at 12,000×g for 10 min at 4 °C. Equal volumes of chloroform:isoamyl alcohol (24:1) were added, the tubes were shaken, centrifuged at 12,000×g for 1 min, and the upper aqueous layer containing DNA was removed. The DNA was precipitated with two volumes of 100% ethanol overnight at −20 °C before pelleting. DNA pellets were washed three times with 70% ethanol, dried, and then resuspended in TE. Quantitation was performed using the Quant-IT DNA quantitation kit (Thermo Fisher Scientific).

### ELISA assays

AGE in plastid and mitochondrial proteins was determined using the OxiSelect™ Advanced Glycation End Product (AGE) Competitive ELISA Kit (Cell Biolabs Inc.; cat # STA-817) as per manufacturer’s instructions. Briefly, equal amounts of plastid or mitochondrial proteins (50 µg) and AGE-BSA standards were added to the wells of the AGE Conjugate coated plate. After washing, anti-AGE antibody (1:1000 dilution) was added to each well and the plate was incubated at room temperature for 1 h. After 3 washes with 1X wash buffer, Secondary Antibody-HRP Conjugate (1:1000 dilution) was added. After 3–5 washes, substrate solution was added, and the reaction was stopped by adding stop solution. Finally, the absorbance of each well was read at 450 nm using a microplate reader. The AGE levels (µg/mL) were calculated based on the AGE-BSA standard as described in the assay manual.

DJ-1 protein in plastids and mitochondria was determined using the Human Park7 Simple step ELISA kit (Abcam; cat # ab215535) as described by the manufacturer. Briefly, plastid or mitochondrial proteins (50 µg) and Park7 standards were loaded into a 96-well plate, Park7 antibody cocktail was added and incubated for 1 h at room temperature. After three washes, TMB (3, 3’, 5, 5’-tetramethylbenzidine) solution was added and absorbance was recorded at 450 nm. The DJ-1 (Park7) protein levels (ng/mL) were determined using the Park7 standards.

AGE in ptDNA and mtDNA was determined using a competitive ELISA assay^23^. Briefly, a microtitre plate was coated with Glycoaldehyde-AGE-BSA (Cell Biolabs Inc.; cat # STA-348) at a concentration of 10 µg/mL in 50 mM sodium carbonate buffer (pH 9.6) overnight at 37 °C. The coupling reaction was discarded and replaced with blocking buffer (2% BSA in 1X PBS). After blocking for 3 h at 37 °C, the plate was washed three times with 1X PBS-T (PBS + 0.05% tween 20). PtDNA or mtDNA samples and AGE-BSA standards were added and mixed with anti-CML antibody (1:100 dilution) (Abcam; cat # ab27684). The competition reaction was incubated for 3 h at 37 °C, the plate was washed 3 times, and horseradish peroxidase coupled anti-rabbit antibodies (1:1000 dilution; ABclonal; cat # AS014) were added and incubated for 1 h. The color was developed with 1-Step™ Ultra TMB-ELISA Substrate Solution (Thermo Fisher Scientific) and the reaction was stopped with stop solution for TMB substrate (Thermo Fisher Scientific). The absorption was read at 490 nm using a plate reader. The AGE levels (µg/mL) were calculated based on the AGE-BSA standard as described^23^.

### Slot-blot assays

AGE in protein and DJ-1 protein slot-blot assays were performed using the Hoefer® Slot blot filtration manifold as per manufacturer’s instructions. Briefly, plastid or mitochondrial proteins (50 µg) were blotted onto a nitrocellulose membrane preincubated in 1X Tris-Buffered Saline (TBS). Equal loading of the total proteins in samples was assessed by staining the membranes with Ponceau S dye as described^45^. Briefly, Ponceau S solution (0.1% Ponceau S in 5% acetic acid) was applied for one minute on membranes. Then the membranes were washed with distilled water until background staining was removed and membranes were photographed. The membrane was blocked overnight at 4º C with 0.1% Tween 20 and 5% BSA in 1X TBS. The membrane was then incubated with anti-AGE antibodies (1:1000 dilution) (Abcam; cat # ab23722) or with anti-DJ-1 antibodies (1:3000 dilution) (Cusabio Technology LLC; cat # CSB-PA03729A0Rb; host-rabbit; specificity-human) for 2 h at 20 °C. The membrane was washed 3 times with 1X TBST (TBS + 0.01% tween 20) and incubated for 1 h with horseradish peroxidase coupled anti-rabbit antibodies (1:10,000 dilution). After incubation, membrane was washed 5 times with 1X TBS-T and developed with 1-Step™ Ultra TMB-Blotting Solution (Thermo Fisher Scientific). The signal intensity of each spot was quantified using ImageJ software (NIH). Additionally, to ensure equal amounts of housekeeping proteins in the samples, the proteins were loaded on the membrane and probed with anti-RbcL antibodies for plastids (1:1000 dilution) (PhytoAB; cat # PHY1413S) and anti-COX2 antibodies for mitochondria (1:1000 dilution) (PhytoAB; cat # PHY0066S).

In order to confirm cross-reactivity of the anti-DJ-1 antibody (Human Park7 antibody from Cusabio) with plant DJ-1 proteins, a western blot was performed using maize and Arabidopsis mitochondrial proteins and including human Park7 protein. See Supplementary Information, Fig. S1.

AGE in DNA slot-blot assays were performed with some modifications^11^. PtDNA and mtDNA samples were denatured in a water bath for 10 min at 100 °C followed by 5 min on ice and blotted (100 ng per slot) onto a nitrocellulose membrane preincubated in 20X saline-sodium citrate (SSC) buffer. After blotting, the membrane was placed on filter paper soaked in denaturation solution (1.5 M NaCl/0.5 M NaOH) for 10 min, followed by soaking in neutralization solution (1 M NaCl/0.5 M Tris–Cl, pH 7.0) for 10 min, and then the membrane was air dried. The membrane was baked for 2 h at 80 °C in an oven with vacuum (25 cm Hg). The membrane was probed with anti-AGE antibodies (1:1000 dilution) (Abcam; cat # ab23722) and developed with 1-Step™ Ultra TMB-Blotting Solution (Thermo Fisher Scientific) as described earlier in the procedure of the ELISA assays. The signal intensity of each spot was quantified using ImageJ software (NIH). DNA amount in each sample was detected by blot-hybridization using mitochondrial specific (cox2) and plastids specific (rbcL) probes. Probes were labeled with DIG labelling mix (Roche). DNA was slot-blotted onto a membrane. The signals representing mtDNA and ptDNA were detected by anti-DIG-alkaline phosphatase conjugates and the NCIP/BCP system (Roche).

### Determination of Amadori products in DNA

Amadori products in ptDNA and mtDNA were determined using the NBT reduction assay^22,23^. The orgDNA samples were mixed with 100 mM sodium carbonate buffer (pH 10.8) containing 0.25 mM nitro-blue tetrazolium (NBT) for 5 h at 37 °C. Absorbance of the reaction solution was read at 525 nm and content of Amadori products was measured using an extinction coefficient of 12,640 cm^-1^ M^-1^ for monoformazon.

### Methylglyoxal (MGO) in organelles

Methylglyoxal amount in plastids and mitochondria was determined using the Methylglyoxal assay kit (Abcam). Briefly, MGO standards were prepared by serial dilution. An equal volume of lysed plastids or mitochondria and standard solutions were added to the reaction mix. The absorbance at 450 nm was measured and MGO levels (nmol) calculated using standard curves. This assay measures “free” MGO as well as MGO in organellar proteins and orgDNA.

### Statistical analysis

All assays were performed at least three times with similar results. The values in each bar graph are shown as mean relative values ± SE from three independent assays (3 biological replicates, each with three technical replicates). Signal intensity of slot-blots was measured using ImageJ and then converted to give the level relative to the tissue with the lowest value, which is set at one. The Mean Relative Intensity ± SE in each assay shows the average of the three biological replicates. The standard error of the ratio (i.e. the Mean Relative Intensity) was determined using the following equation:

$${\varvec{S}}{\varvec{E}}\left(\frac{{\varvec{x}}}{{\varvec{y}}}\right)=\left(\frac{{\varvec{x}}}{{\varvec{y}}}\right)\sqrt{{\left(\frac{{\varvec{S}}{\varvec{E}}\left({\varvec{x}}\right)}{{\varvec{x}}}\right)}^{2}}+{\frac{{\varvec{S}}{\varvec{E}}\left({\varvec{y}}\right)}{{\varvec{y}}}}^{2}$$

where x, SE(x), y, and SE(y) are the Mean value (signal intensity) and standard error from three assays.

Statistically significant differences between tissues were assessed by the ANOVA, and Tukey honest significant difference test and are shown as asterisks, where *P-value ≤ 0.05, **P-value ≤ 0.01, ***P-value ≤ 0.001 are indicated on respective graphs.

## Supplementary Information

Supplementary Information.
