# Supplementary material for: Glycation damage to organelles and their DNA increases during maize seedling development
Source: Sci Rep. 2022 Feb 17;12:2688. doi: 10.1038/s41598-022-06454-7 (PMC8854438; doi:10.1038/s41598-022-06454-7)
Supplement: Supplementary file 1 — Supplementary Information. [file 41598_2022_6454_MOESM1_ESM.pdf]

Supplementary Information

Title: Glycation damage to organelles and their DNA increases during maize seedling development

Running title: Organelle damage during maize development

Authors: Diwaker Tripathi, Delene J. Oldenburg, and Arnold J. Bendich*

Affiliation: Department of Biology, University of Washington, Seattle, WA, United States

***Correspondence:**
Arnold Bendich
(bendich@uw.edu)

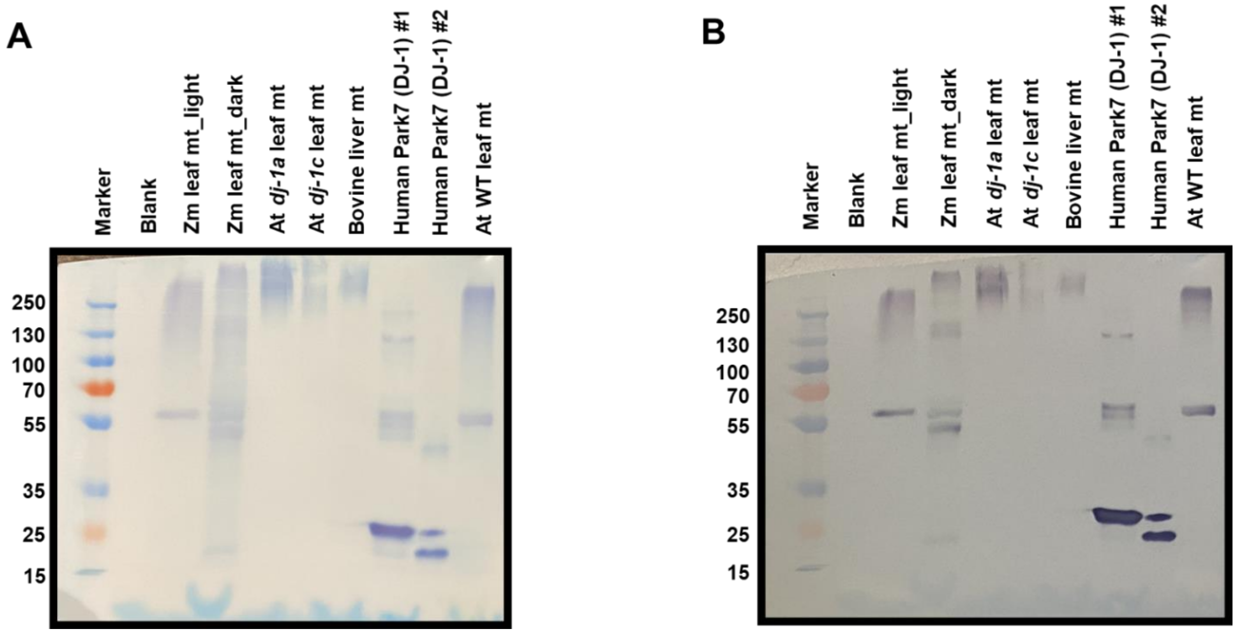

Figure S1 – (A). Size fractionation of DJ-1 mitochondrial proteins in plants detected with human DJ-1 antibody. Total mitochondrial proteins and recombinant human Park7 protein were separated by SDS-PAGE. After electrophoresis, proteins were transferred to a membrane and probed with human anti-DJ-1 antibody and developed with TMB substrate. lane 1: size markers (in kDa); lane 2: blank; lane 3: maize mitochondrial protein from leaves grown in light (Zm leaf mt_light); lane 4: maize mitochondrial protein from leaves grown in dark (Zm leaf mt_dark); lane 5: Arabidopsis mitochondrial protein from leaves of *dj-1a* mutant line; lane 6: Arabidopsis mitochondrial protein from leaves of *dj-1c* mutant line; lane 7: bovine mitochondrial protein; lanes 8 and 9: human Park7 proteins (#1 and #2); lane 10: Arabidopsis mitochondrial protein from leaves grown in light. (B). Longer exposure of the blot used in Fig. S1(A).

Supplementary Methods

Seeds of *Arabidopsis thaliana* wild-type Columbia-0 (Col-0) and sequence-indexed T-DNA insertion lines of DJ-1A (SALK_125939) and DJ-1C (SALK_125439) were obtained from the Arabidopsis Biological Resource Centre (https://abrc.osu.edu/stocks). Maize [*Zea mays* (L.), inbred line B73] and Arabidopsis seeds were sown in Sunshine soil Mix #4 and vermiculite (1:1 ratio). Maize seedlings were grown for 12 days with a 16 h light/8 h dark photoperiod (light-grown) or in continuous dark for 12 days (dark-grown) in a temperature-controlled room. Similarly, wild-type Col-0 and dj-1 mutant seeds were grown for 4-weeks with a 16 h light/8 h dark photoperiod. Seedlings were washed with 0.5% sarkosyl for ~3 min and then rinsed with distilled water. For each assay, tissue was harvested from 30-50 plants. The plant mitochondrial proteins were isolated as described in the Methods section. The supernatant was collected, and total protein amount was quantified with a Pierce™ BCA Protein Assay kit (Thermo Fisher Scientific). The bovine mitochondrial proteins were isolated from liver tissue (purchased from a local butcher store) by Mitochondria Isolation Kit for Tissue (Thermo Scientific). As positive controls, approximately 100 ng of two different Recombinant Human Parkinson disease protein 7 (Park7) preparations were used: #1 Park7; Full+ 36 aa His-Tag (24 kDa) from RayBiotech (cat # 228-11203-2); and #2 Park7-Partial (19.8 kDa) from Cusabio Technology LLC (cat # CSB-EP860342HU1e1). Twenty-five ug of plant mitochondrial proteins and 10 ug of bovine mitochondrial proteins were mixed with 4X SDS-PAGE gel. The mixtures were heated at 95-100° C and loaded onto a 10% SDS-PAGE gel. The gel was run at 40-60 V for 2 hr. Afterwards, the gel was transferred onto the PVDF membrane. The membrane was blocked with 5% non-fat milk+1X TBS-T for 1 hr. After blocking, the blot was probed with 1:3000 human anti-DJ-1 antibody (Cusabio Technology; cat # LLC-CSB-PA03729A0Rb) for 2 hr at room temperature. Next, blots were washed with 1X TBS-T and incubated for 1 hr with horseradish peroxidase coupled anti-rabbit antibodies (1:10000 dilution). After incubation, the membrane was washed 5 times with 1X TBS-T and developed with 1-Step™ Ultra TMB-Blotting Solution (Thermo Fisher Scientific). Molecular mass (in kDa) markers were from PageRuler™ Plus Prestained Protein Ladder (Thermo Fisher Scientific).

Supplementary Results

Several human DJ-1 homologs have been predicted in maize and some DJ-1 homologs have been identified in Arabidopsis. Arabidopsis DJ-1 proteins are predicted to have sizes between 41-51 kDa and some are found in plastids and mitochondria[1]. All plant DJ-1 homologs analyzed exhibit 30-40% similarity in amino acid sequence with human DJ-1 protein and have several conserved protein binding sites[2]. In our western blot (Fig. S1), we found a DJ-1 band at approximately 55 kDa in mitochondria from leaf tissues of maize and Arabidopsis grown in light, while there was more than one band in mitochondria from leaf tissues of maize grown in dark, suggesting that dark-grown plants have DJ-1 homologs of different sizes. Note the faint band in dark-grown maize mitochondria (lane 4) that corresponds to the human Park7 protein at 19.8 kDa. As expected, we did not find DJ-1 bands in mitochondrial proteins from two different lines of At *dj-1* mutant plants (lanes 5, 6). Additionally, we examined another mammal (bovine) to test reactivity of human DJ-1 antibody. We found a faint ~25 kDa DJ-1 band in bovine liver mitochondria suggesting the presence of DJ-1 in bovine liver tissue. We conclude that human DJ-1 antibody recognizes both plant and human DJ-1 proteins.

References

1. Ghosh, A. et al. Presence of unique glyoxalase III proteins in plants indicates the existence of shorter route for methylglyoxal detoxification. *Scientific Reports* **6**, 18358 (2016).

2. Xu, X.M. et al. The Arabidopsis DJ-1a protein confers stress protection through cytosolic SOD activation. *J Cell Sci* **123**, 1644-1651 (2010).
